# Protective Effects of Crocin Against Hepatic Damages in D-galactose Aging Model in Rats

**DOI:** 10.22037/ijpr.2019.15022.12825

**Published:** 2020

**Authors:** Seyedeh Farzaneh Omidkhoda, Soghra Mehri, Somaye Heidari, Hossein Hosseinzadeh

**Affiliations:** a *Department of Pharmacodynamics and Toxicology, School of Pharmacy, Mashhad University of Medical Sciences, Mashhad, Iran. *; b *Pharmaceutical Research Center,* *Pharmaceutical Technology Institute, Mashhad University of Medical Sciences,* *Mashhad, Iran.*

**Keywords:** D-galactose, Crocin, Saffron, Aging, Lipid peroxidation, iNOS

## Abstract

Aging is a progressive process which is associated with liver dysfunction and it is due to oxidative stress, inflammation, and cell apoptosis. Long-term D-galactose (D-gal) administration is able to develop an aging model in animals. Crocin as a major active ingredient in saffron has shown anti-inflammatory and hepatoprotective effects via its antioxidant capacity. Thus, the aim of the present study was the assessment of crocin effects on hepatic and metabolic disorders induced by D-gal in rats. Aging model was induced in rats by 56-day administration of D-gal (400 mg/kg/day subcutaneously). Protective effects of different doses of crocin (7.5, 15 and 30 mg/kg/day) in concomitant with D-gal administration were evaluated. Malondialdehyde (MDA) and reduced glutathione (GSH) amounts were measured by means of their reaction, respectively, with thiobarbituric acid and 5,5′-Dithiobis (2-nitrobenzoic acid) (DTNB) under a specific condition. Cyclooxygenase-2 (COX-2), β-galactosidase, induced nitric oxide synthase (iNOS), and carboxymethyllysine (CML) levels were determined by western blotting method. Additionally, the levels of alanine aminotransferase (ALT), aspartate aminotransferase (AST), and alkaline phosphatase (ALP) were measured in serum. D-gal administration significantly elevated ALT, AST, ALP levels, which were markedly inhibited by crocin administration. Crocin suppressed the overgeneration of lipid peroxidation products such as MDA. iNOS was elevated by D-gal administration and was returned to the normal extent by crocin. Therefore, Crocin as a powerful antioxidant and radical scavenger, totally exhibited hepatoprotective effects against D-gal-induced toxicity in rats.

## Introduction

Aging is a progressive and gradual process that is associated with decreased quality of physiological functions in organisms and subsequently leads to tissue dysfunction, diseases, and death (1). Numerous studies have shown various mechanisms which are responsible for aging development or expediting (2). The most important considered reason for aging is oxidative stress and overproduction of free radicals such as reactive oxygen and nitrogen species (ROS and RNS) (3). Some investigations reveal increased induced nitric oxide synthase (iNOS) expression and peroxynitrite in aging situation (3, 4). Oxidative stress can be associated with various harmful consequences. Apoptosis and then tissue necrosis can be induced by oxidative stress (5). Moreover, free radicals are able to activate innate immune system response (6). Inflammation also amplifies the generation of ROS and RNS, conversely. Free radicals have mutagenic effects on organism’s genome after a chronic inflammatory situation (1). Another result of oxidative stress is lipid peroxidation producing malondialdehyde (MDA) as a major reactive output (7), while, there are fewer conflictive results of alteration in lipid peroxidation products in the young to aged animals with different species, sexes, strains, and examined organs (1). During aging development, the alleviation of enzymatic or non-enzymatic antioxidant system components such as glutathione (GSH), superoxide dismutase (SOD), and catalase (CAT) has been observed, as well (8). Other changes including elevated cyclooxygenase-2 (COX-2) expression and NF-κB activity in cell lysosomes have also been reported (9). In addition, it has been indicated that in aging and age-related diseases, protein glycation and afterward advanced glycation end-products (AGEs), especially carboxymethyllysine (CML) level increase. These compounds can induce and provoke oxidative stress phenomenon (10).

Chronic D-galactose (D-gal) administration, during 6-8 weeks, develops a valuable aging model that is accompanied by biochemical, histopathological, and even outward changes in rats or mice. Abundant original studies which are directly or indirectly related to D-gal have widely proposed aging-like alterations in animals (11-15). 

D-gal as the same as physiological senescence can be concomitant with diabetic effects (2, 16). Results acquired from an experimental study, demonstrated that during aging progress, insulin resistance increased and accordingly calcium sensing receptor (CaSR) expression and glucose-dependent insulin secretion were significantly raised (17). Some evidences represent aggravating effects of oxidative stress on metabolic disorders such as diabetes (18).

Crocin is one of the main bioactive ingredients of saffron, which is prepared from *Crocus sativus *L*. *plant’s stigmas. It hasbeen shown different traditional therapeutic usages in various disorders, including inflammation, diabetes, cancer, bronchitis, and skin disorder (19). Crocin also possesses antioxidant effects in different studies and has increased GSH level, SOD, and CAT expression and has decreased MDA, NOS, nitric oxide (NO), and basically ROS production (20, 21). This component is also capable of free radical scavenging (22), anti-inflammatory, and hepatoprotective potential (23). An original study claims that crocin resulted in inhibition of apoptosis, reduction of alanine aminotransferase (ALT), aspartate aminotransferase (AST), alkaline phosphatase (ALP) level, and prevention of DNA fracture (24). This component has also shown the protective effects on methyl methanesulfonate-induced DNA lesions in liver, lung, kidney, and spleen (25). Furthermore, crocin could suppress iNOS, COX-2, interleukin β (IL-β), and tumor necrosis factor α (TNF-α) expression in cell culture assessment by upregulation of CX3C chemokine receptor 1 (CX3CR1) which causes inactivation of microglial cells (26). It has been demonstrated that crocin is able to prevent apoptosis induced by diazinon in hepatocytes via reduction of caspases-9 and -3 activation and Bax/Bcl-2 ratio (27). Crocin exhibited anti-metabolic syndrome effects that exerts favorable effects on diabetes. It has been shown that crocin is able to reduce blood glucose, HbA1c, and increase insulin level (28).

Shahroudi *et al*. (2017) found that the fixed oil of *Nigella sativa* had the ameliorating influence of aging-associated changes in the brain and liver of mice induced by D-gal (500 mg/kg, SC)) (29). A similar study was conducted by Mohammadi *et al*. (2017) showed that crocin could prevent damages arising from aging developed by D-gal administration in mice (30). Therefore, based on above-mentioned explanations, we assessed the effect of crocin on aging, hepatic, and metabolic disorders induced by D-gal in rat liver.

## Experimental

*Materials*

Malondialdehyde tetrabutylammonium (MDA) and reduced glutathione (GSH) were obtained from Sigma. We also purchased rabbit monoclonal anti-Cox2 antibody from Cell Signaling, rabbit polyclonal anti-iNOS and anti-CML antibody from Abcam, rabbit purified anti-β-galactosidase antibody from Biovision, and mouse monoclonal anti-GAPDH from Abcham. Polyvinylidene fluoride (PVDF) membrane was obtained from Bio-Rad. 

*Crocin preparation*

Stigmas of *C. sativus* L. in Iridaceae ‎family were purchased from Novin Saffron, Iran. Crocin was extracted and purified by crystallization method based on the previous study. This method was carried out at different temperatures in the first and second steps, and ethanol 80% was used as the extraction solvent. The purity of crocin in this method is more than 97% (31).

*Animals*

Male Wistar rats were used for this study, with a weight range of 230-250 g. They were kept in the colony room of pharmacy faculty of Mashhad university of medical sciences, Iran. The conditions were 12/12 h light/dark cycle at the 21±2 ºC temperature and free access to food and water. All animal experiments were conducted in accordance with Ethical Committee Acts of Mashhad University of Medical Sciences (code: 950536). 

*Study design and treatments*

Aging was induced by administration of D-gal (400 mg/kg/day), subcutaneously (SC) during 56 days. In the current study, to investigate the effect of crocin on this model, 6 groups of the animals were designed with different interventions that include: 

(1) Control group (treated with normal saline, SC) 

(2) D-gal (400 mg/kg/day, SC) 

(3) Crocin (30 mg/kg/day Intraperitoneally (IP)) 

(4) D-gal (400 mg/kg/day SC) + Crocin (7.5 mg/kg/day IP) 

(5) D-gal (400 mg/kg SC) + Crocin (15 mg/kg/day IP) 

(6) D-gal (400 mg/kg SC) + Crocin (30 mg/kg/day IP) (32, 33)

*Liver tissue and serum preparation*

At the end of treatment, the rats were sacrificed and their liver tissues, after washing with normal saline, were completely removed. Then a part of tissues were immediately cut and put in liquid nitrogen and finally stored at −80 °C until use. At the same time, their blood was collected and centrifuged at 4000g for 15 min to obtain the blood serum and then stored at −80 °C.

*Lipid peroxidation assay*

The level of MDA was measured as the marker of lipid peroxidation. Briefly, 180-200 mg of the frozen samples were weighted and homogenized in 1.15% W/V KCl solution to have 10% tissue homogenates. Then, 3 mL phosphoric acid (1%) and 1 mL thiobarbituric acid (0.6%) were added to 0.5 mL of tissue homogenates to produce a pink colored complex with tissue MDA. In the next step, the mixtures were heated for 45 min in a boiling water bath and after cooling, 4 mL of n-butanol were added to each of the samples and mixed vigorously for 1 min. The mixtures were centrifuged at 4000 g for 20 min. Finally, the organic layers were transferred to a fresh tube and their absorbance was recorded at 532 nm (34).

In order to calculate the amounts of MDA in the samples, a calibration curve was plotted by using MDA tetrabutylammonium. The MDA levels have been expressed as nmol/g tissue.

*Reduced glutathione (GSH) assay*

For this assay, we used the method of Moron *et al*. (1979) with minor differences. Briefly, after weighting and homogenization of the tissues in sodium phosphate buffer (pH = 7), 0.25 mL of the homogenates was added to 0.25 mL of 10% trichloroacetic acid (TCA) and mixed strongly. After centrifuging at 10000 g for 7 min at 4 °C, the supernatants were mixed with 2 m phosphate buffer (pH = 8) and 0.5 mL of DTNB (5,5′-Dithiobis(2-nitrobenzoic acid))/sodium citrate solution. Then, the absorbance of the samples was measured at 412 nm by means of a spectrophotometer (Jenway 6105 UV/vis, UK) during 10 min (35). GSH contents were calculated by a standard curve produced using commercially available GSH. Levels of GSH have been expressed as nmol/g tissue.

*Western Blotting*

For western blot analysis, the samples were lysed in lysing buffer which contained 50 mM Tris-HCl (pH = 7.4), 2 mM EDTA, 2 mM EGTA, 10 mM NaF, 1 mM sodium orthovanadate (Na3VO4), 10 mM β-Glycerophosphate, 0.2% W/V sodium deoxycholate, 1 mM phenylmethylsulfonyl fluoride (PMSF), and complete protease inhibitor cocktail (Sigma P8340). The total proteins were separated on SDS-PAGE gels with appropriate concentration (for COX-2 and CML assessment 12%, for iNOS and β-galactosidase assessment 10%) and transferred to polyvinylidene fluoride (PVDF) membranes. Then, the blots were blocked with skim milk (5% during 2 hrs for all, with exception of CML that was blocked with 2% skim milk for an hr) at room temperature. After blocking, the blots were incubated with primary antibodies: anti-Cox-2 (#12282), anti-iNOS (ab3523), anti-β-galactosidase (3231-100), anti-CML (ab27684), anti-β-actin (cell signaling #4967) and anti-GAPDH (#ab8245) 1000-fold dilutions (just for anti-β-galactosidase with a dilution of 1/600) for 2 hrs at room temperature (but for CML detection, the page was incubated with primary antibody for 16-18 h in 4 °C). Then the membranes were washed three times with 0.1% Tween 20 and TBST, and then incubated with horseradish-peroxidase conjugated anti-rabbit antibody (#7074 Cell Signaling) at 1:3000 dilutions for 90 min at room temperature. Finally, the protein bands were detected using enhanced chemiluminescence (ECL) reagent and Alliance 4.7 Geldoc (UK) software. The protein bands were analyzed using UVtec software (UK). The protein levels were normalized against corresponding β - actin or GAPDH intensities.

*Biochemical serum tests*

The levels of biochemical biomarkers, including aspartate transaminase (AST), alanine transaminase (ALT), alkaline phosphatase (ALP), glucose, and insulin were evaluated using standard diagnostic kits with a clinical spectrophotometer (Hitachi, Japan).

*Statistical Analysis*

The results are expressed as mean ± SD. Statistical analyses were performed using one – way ANOVA followed by Tukey–Kramer test to compare the differences between the means. The differences were considered statistically significant when *P* < 0.05.

## Results

*Effect of crocin on D-gal-induced hepatotoxicity in rats*

**Figure 1:**
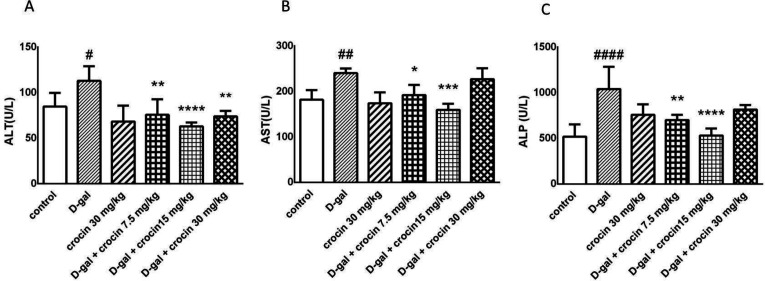
Effects of D-gal and crocin administration on ALT **(A)**, AST **(B)** and ALP **(C)** level. The data are displayed as mean ± SD (n = 4). #*P *< 0.05, ##*P *< 0.01 and ####*P *< 0.0001 *vs*. control group; *P < 0.05, **P < 0.01,***P < 0.001 and ****P < 0.0001 *vs*. D-gal-treated group. D-gal: D-galactose

Administration of D-gal significantly elevated the level of liver enzymes ALT (*P *< 0.05), AST (*P* < 0.01) and ALP (*P* < 0.0001) as compared to control. Liver function tests showed significantly decreased ALT, AST, and ALP levels by crocin treatment in comparison with the D-gal group. In all three tests, crocin at the dose of 15 mg/kg/day had the best effects (Figure 1)

*Effect of crocin and D-gal on the level of insulin and blood glucose*

**Figure 2:**
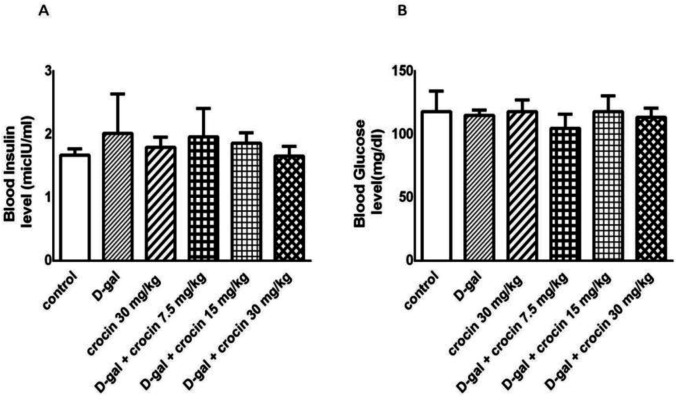
**Effects of D-gal and crocin administration on** **Insulin **(A)** and blood sugar **(B)** level. The data are expressed as Mean ± SD (n = 5). D-gal: D-galactose**

The results of blood glucose and serum insulin level evaluation indicated that D-gal slightly increased insulin, but had approximately no effects on blood glucose. Crocin also attenuated insulin and blood glucose level, but these effects were not significant (Figure 2).

*Effect of crocin on lipid peroxidation induced by D-gal in the liver*

**Figure 3:**
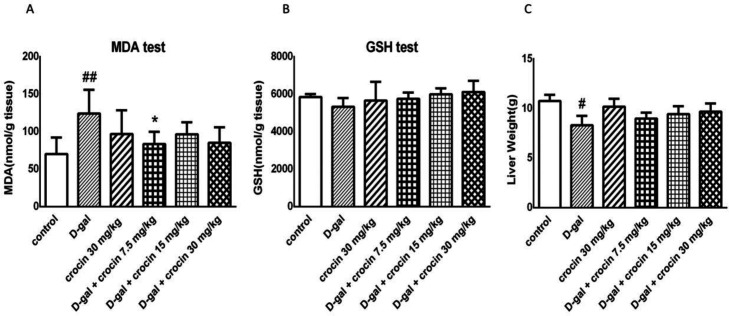
**Effects of D-gal and crocin administration on** **MDA **(A)**, GSH **(B)** levels and liver weight **(C)**.** The data are expressed as Mean ± SD (n = 4). ##*P *< 0.01 *vs*. control group; *P < 0.05 *vs*. D-gal-treated group. D-gal: D-galactose

As shown in (Figure 3A) D-gal significantly elevated MDA generation as a lipid peroxidation product when compared to control (*P* < 0.01). Crocin, especially at dose 7.5 mg/kg/day, suppressed MDA overproduction (*P* < 0.05 *vs*. D-gal-treated group). Also, our results represented that GSH level diminished in the D-gal-treated group and returned to the normal state by crocin administration, but not significantly (Figure 3B). In addition, D-gal decreased liver weight significantly while concomitant administration of crocin increased liver weight, but it was not statistically significant (Figure 3C).

*Effect of crocin and D-gal on the protein level of iNOS*

**Figure 4:**
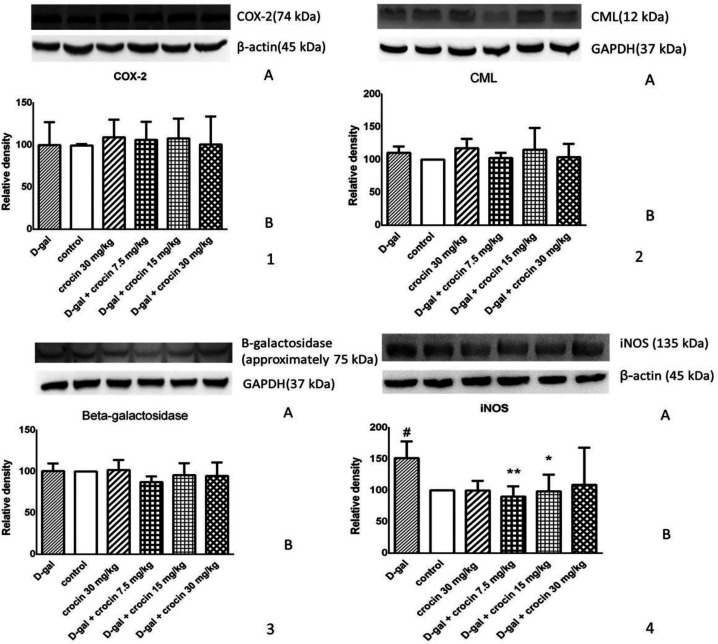
**Effect of crocin and D-gal on the protein levels of (1**)** COX-2, (2**) **CML,** and (3) β-galactosidase. (1A), (1B) and (1C) are western blot images of COX-2, CML and β-galactosidase, respectively. (2A), (2B) and (2C) are determined using densitometric analysis. The data are presented as Mean±SD (n = 4). D-gal: D-galactose. (4) Effect of crocin and D-gal on the protein level of iNOS. (4A) is western blot images of iNOS and β-actin. (4B) is determined using densitometric analysis. The data are presented as Mean ± SD. n = 4. #*P *< 0.05 *vs*. control group; *P < 0.05, **P < 0.01 *vs. *D-gal-treated group

The level of iNOS protein was significantly raised in the liver following administration of D-gal (*P* < 0.01 *vs*. control), while treatment with crocin (7.5 and 15 mg/kg/day) markedly reduced the level of iNOS protein to the extent of the normal content. The results have been shown in Figure 4 in parts (4A) and (4B).

*Effect of crocin and D-gal on the protein levels of COX-2, CML, and β-galactosidase*

With regard to Figure 4 (1, 2, 3), our data did not indicate any excessive amounts of COX-2, CML and β-galactosidase proteins in the rat liver. In comparison with the others, the CML level had more visible changes, as in the D-gal group is a little higher than the control group, while with 7.5 mg/kg of crocin administration, the CML has become lower, but not statistically significant.

## Discussion

This study evaluated protective effects of crocin against D-gal-induced hepatotoxicity. Crocin prevented the liver damage that it is shown by ALT, AST, and ALP markers. The excessive release of these enzymes was significantly inhibited by crocin administration. Crocin suppressed the overgeneration of lipid peroxidation products such as MDA. iNOS was elevated by D-gal administration and was returned to the normal extent by crocin. 

The most commonly mentioned mechanism for the development of the aging model by chronic D-gal administration is oxidative stress, ROS accumulation, and subsequent destructive events (11-16, 36). Taken together, we believe that crocin as a powerful antioxidant and radical scavenger (21), reversed all aging-like effects which were induced by D-gal administration in some biological aspects. Its evidence is decreased level of biochemical factors representing liver function such as ALT, AST, ALP, and also suppression of lipid peroxidation and MDA overproduction. Our findings demonstrate that in contrast to general expectation and some previous-mentioned experimental studies, GSH level did not have a significant change in the liver due to D-gal oversupplying. Hadzi-petrushev *et al*. (2015) assessed the alterations of antioxidant enzymes induced by D-gal in different ages in liver and kidney of rats. They suggested that hepatic catalase (CAT) and glutathione peroxidase (GPx) activity, were even raised in all age groups of rats and glutathione reductase (GR) activity possessed approximately no difference between control and D-gal group in elderly rats. The increment of antioxidant activity is known as an adaptive response to neutralizing oxidant agents (37). The findings, obtained from the study done by Samarghandian *et al*. (2016) demonstrated that CAT enzyme level in kidney of the rats with 10 months old did not change and crocin was not able to increase CAT, SOD, and GPx significantly in these animals but the alterations in rats with 20 months old was considerable (38). On the other hand, in Kasapoglu and Ozben study (2001), dispensability of aging-related reduction in antioxidant capacity was established (39). In our study, enzymatic antioxidants rather than GSH may have changed due to D-gal administration.

There is a body of convincing evidence that oxidative stress and free radicals play an essential role in obesity promotion, insulin resistance, and subsequently diabetes (18). Oh *et al*. (2016) showed that in aged mice, insulin sensitivity decreased and glucose-dependent insulin secretion increased through overexpression of Ca sensing receptors. It should be considered that in this study, non-fasting blood glucose level did not change with age in 4, 8, 12 and 20 months old mice. These findings give us insight into the fact that aging does not always lead to hyperglycemic status (17). Streptozocin as a diabetogenic agent acts via induction of oxidative stress and reduction of brain-derived neurotrophic factor (BDNF) in pancreas, brain, and liver (40). In an experimental study crocin had protective effects on insulin resistance and hyperlipidemia induced by Streptozocin in the rats (41). These approaches made us curious about D-gal that whether it has effects on blood glucose and insulin (through ROS generation) and also the effects of crocin on these likely metabolic changes. There are some *in-vivo* studies in which galactose was used to generate diabetic cataract model in animals such as dogs or rats. However, galactose dosage and duration of its administration was more than our study (42-44). In our experiment, with a 56-day injection of D-gal (400 mg/kg/day), no insulin resistance was observed. Similar to our study, song *et al*. (1999) indicated that D-gal (50 mg/kg/day SC) failed to cause diabetes model in mice, probably because of its different mechanisms responsible for aging simulation rather than diabetes induction (16). 

As our data represent, iNOS overexpression was inhibited by crocin administration. Nitric oxide can participate in a reaction with superoxide to generate reactive oxygen and nitrogen species. indeed, the most important point to drive ROS production and aging process is the NO/superoxide imbalance. It has also been exhibited that NO have reversible hampering effects on cytochrome c oxidase of mitochondria, that subsequently leads to less O_2_ consumption and ROS formation. Based on this inference, some researchers believe that decreased NO level is a trigger for aging, thus reduction in NO level will be followed by increment of O_2_ consumption and ROS production (3, 45). Our findings support some investigations that have the same results as ours (4, 26, 46).

It was reported that β-galactosidase is one of the most popular biomarkers for detection of senescent cells (2). Basically, senescence associated β-galactosidase (SA-β-gal) activity is measured by staining of the cells or tissues with a chromogenic agent, but its level can be determined via western blotting as well, although its alterations are less observable by this method. The study was conducted by Lee *et al*. (2006) demonstrated that the origin of SA-β-gal is the cell lysosome and the variations in SA-β-gal is a result of the changes in the number or activity of these organelles. The elevation of the β-galactosidase level is due to the aging process and the stop in cell replication, not the reason of that. Therefore, after discontinuity of the cell proliferation and accumulation of inactive or dead cells in tissue, the amounts of lysosomes and their enzymes will rise (9). In regard to recent explanation, we can conclude that if aging process induces apoptosis in cells, the β-galactosidase may even decrease its level. Furthermore, it has been shown that the long term D-gal administration can result in the β-galactosidase rise but with some different methods from that of our investigation. In Ruan *et al.* study (2013), the method of detection was tissue staining (15). In the other study, which was carried out by Huang *et al*. (2013), aging model was induced by D-gal intraperitoneal injection for 12 weeks (150 mg/kg/day) and the time of exposure was longer than that of our study (36). Our treatment (with higher dose and 4 weeks shorter duration of D-gal injection) might cause more cell apoptosis and so, had no significant changes in β-galactosidase level.This hypothesis is also supported by liver weights as well. The decrement of liver weights in the D-gal group is possibly due to tissue necrosis and cell apoptosis as shown in some histomorphological assessments (14). 

Another complication in the aging phenomenon is the generation of advanced glycation end-products (AGEs) such as CML that can lead to the various harmful outcomes, for example predisposing to diabetes, neurodegenerative disorders, rheumatoid arthritis (10), cardiovascular events, and arterial stiffness. This CML rise is significantly higher in elderly human than the old rats, because of their faster protein turnover and shorter lifetime (8). The excessive production of CML during D-gal-induced aging has been demonstrated in cell culture and animal models (16, 47). In our experiment, similar results to previous studies were obtained, but were not significant. 

Cyclooxygenase-2 (COX-2) is mostly an inducible isoform of COX enzyme in the inflammatory condition, which is responsible for the synthesis of prostaglandins (PGs) from arachidonic acid. These molecules play an imperative role in both physiological and pathological pathways. A systematic study was performed by Kirkby *et al*. (2016) showed that constitutive COX-2 also exists, with the greatest level of expression in the uro-renal, gastrointestinal and central nervous systems (48). Oxidative stress can connect to the inflammation and immune system via Toll-like receptors (6) and lipid peroxidation is able to activate the components of immune system, too (7). The most common regulatory agent for COX-2 expression is NF-κB transcriptional factor (48). As it is reported by Poligone and Baldwin (2001), NF-κB activity increases via several signaling factors and induces COX-2 expression. This enzyme produces PGE_2_ in the early stage of inflammation and PGJ_2_ and PGA in the late stage of inflammation. Therefore, at first, PGE_2_ leads to more NF-κB level and subsequent events such as IL-8 expression, but finally, PGJ_2_ and PGA enhancement creates a negative feedback on NF-κB activity and causes COX-2 suppression (49). In some previous studies, COX-2 level was assessed after induction of aging model by D-gal in mouse kidney (46), mouse liver (50), rat brain (51), and rat prostate (52) and their findings exhibited that COX-2 level elevated in comparison with the control group. Yan *et al*. (2016) simulated acute aging model in rat liver by intraperitoneal injection of 20 μg/kg of LPS and after 15 min, 700 mg/kg of D-galactosamine. They observed that COX-2 level in the aging-model group increased (53). In our study, surprisingly, D-gal administration (400 mg/kg/day, for 56 days, SC) did not significantly change the COX-2 level in rat liver. 

In conclusion, decreased level of ALT, AST, and ALP level by crocin in D-gal-treated rats revealed that this valuable compound has protective effects on hepatocytes. Crocin as a powerful antioxidant and radical scavenger was able to decrease MDA, a major product of lipid peroxidation, and inhibit iNOS and subsequently RNS overproduction which is associated with the aging process. Therefore, it can be considered for therapeutic purposes or as a useful supplement.
